# Altered Estrogen Receptor Signaling Pathway in BRCA2‐Deficient Estrogen Receptor‐Positive/HER2‐Negative Breast Cancer

**DOI:** 10.1002/cnr2.70558

**Published:** 2026-04-24

**Authors:** Kaori Kawasaki, Misato Masuyama, Masafumi Shimoda, Ikumi Seto, Chieko Mishima, Yoshiaki Sota, Kaori Abe, Nanae Masunaga, Masami Tsukabe, Tetsuhiro Yoshinami, Tomohiro Miyake, Tomonori Tanei, Kenzo Shimazu

**Affiliations:** ^1^ Department of Breast and Endocrine Surgery Osaka University Graduate School of Medicine Suita Osaka Japan

**Keywords:** *BRCA* mutations, breast cancer, estrogen receptor alpha, phosphorylation, signal transduction, translational research

## Abstract

**Background:**

Hereditary breast cancer accounts for approximately 10% of all breast cancer cases, with germline *BRCA2* pathogenic variants (PVs) being the most prevalent genetic alteration. *BRCA2* PVs predominantly lead to estrogen receptor (ER)‐positive/HER2‐negative breast cancers, which exhibit more aggressive phenotypes compared to sporadic cases. However, the specific effect of *BRCA2* deficiency on ER signaling remains poorly understood.

**Aims:**

This study aimed to elucidate the relationship between *BRCA2* deficiency and ER signaling using integrated clinical and in vitro analyses.

**Methods and Results:**

Immunohistochemical analyses were performed on ER‐positive/HER2‐negative breast tumors from *BRCA2* PV carriers (*n* = 8) and *BRCA2* wild‐type patients (*n* = 59). Furthermore, two *BRCA2*‐deficient ER‐positive/HER2‐negative MCF7 cell lines were generated using CRISPR‐Cas9 with two distinct guide RNAs targeting *BRCA2*, followed by Western blotting and functional assays. Immunohistochemical analyses demonstrated significantly lower levels of phosphorylated (p)‐ER Ser167, p‐AKT Ser473, and RB1 in *BRCA2* PV carriers compared to patients with *BRCA2* wild‐type (*p* = 0.002, 0.018, and 0.037, respectively). Western blotting confirmed reduced p‐ER Ser167, p‐AKT Ser473, and RB1 levels in *BRCA2*‐deficient cells relative to parental MCF7 cells. Decreased ER and AKT phosphorylation was not associated with consistent changes in the expression levels of downstream estrogen‐responsive genes or proteins. Functional assays revealed that *BRCA2* deficiency significantly increased sensitivity to the poly (ADP‐ribose) polymerase inhibitor olaparib, whereas tamoxifen sensitivity remained unchanged.

**Conclusion:**

This study presents the first detailed characterization of ER signaling alterations in ER‐positive/HER2‐negative breast cancers with germline *BRCA2* PVs, offering insights for the development of targeted therapeutic strategies for this patient population.

## 1 Introduction

Nearly 10% of breast cancers are hereditary, with 50% involving pathogenic variants (PVs) in the germline *BRCA1* or *BRCA2* genes [1]. Women with germline *BRCA1* or *BRCA2* PVs have a cumulative breast cancer incidence exceeding 70% by the age of 80 [2]. These genes play critical roles in the DNA damage response (DDR) pathway [3]. BRCA1 is recruited to sites of DNA damage and activates the DNA helicase SMARCAD1 to promote double‐strand break (DSB) site excision. In addition, BRCA1 activates PALB2, which recruits BRCA2 to DSB sites. BRCA2 works with PALB2 to facilitate recombinase RAD51 recruitment to single‐stranded DNA, a key step in error‐free homologous recombination. Deleterious mutations in *BRCA1*, *BRCA2*, or both cause cells to depend on error‐prone DNA repair mechanisms that rely on poly (ADP‐ribose) polymerase (PARP). As a result, breast cancer cells with *BRCA1* or *BRCA2* PVs are sensitive to PARP inhibitors such as olaparib.

Although *BRCA1* and *BRCA2* PVs share similarities, the breast cancer subtypes they cause are different. Approximately 70% of *BRCA1* PV carriers develop triple‐negative breast cancer, whereas nearly 80% of *BRCA2* PV carriers develop estrogen receptor (ER)‐positive, HER2‐negative breast cancer [4]. This difference in ER positivity stems from distinct molecular effects: *BRCA1* deficiency suppresses estrogen signaling and DDR pathways, whereas *BRCA2* deficiency solely affects DDR, leading to genomic instability and increased accumulation of DNA damage due to estrogen stimulation in breast epithelial cells [5, 6, 7, 8].

*BRCA2* deficiency contributes to the aggressiveness of ER‐positive breast cancer. ER‐positive/HER2‐negative breast cancers are classified into luminal A and B subtypes. The luminal B subtype is more aggressive, with low endocrine therapy sensitivity compared with luminal A [9]. Although luminal A and B subtypes occur equally in sporadic breast cancer, the luminal B subtype is 5 times more prevalent in *BRCA2* PV carriers [10]. This aggressive phenotype is linked to disrupted cell cycle regulation, particularly at the G2/M checkpoint and during S‐phase progression [11, 12]. Furthermore, Oncotype Dx analysis, which predicts response to endocrine therapy, indicates a higher risk of recurrence in *BRCA2* PV‐associated breast cancer compared to sporadic cases [13]. In addition, lymph node metastasis is more common in *BRCA2* PV‐associated breast cancer, indicating a poorer prognosis for these patients [14].

*BRCA2* PV‐associated ER‐positive breast cancer may exhibit resistance to endocrine therapy and poorer outcomes compared to sporadic cases, reflecting the reduced therapy sensitivity of luminal B breast cancer. However, the effectiveness of endocrine therapy and prognosis in *BRCA2* PV‐associated ER‐positive breast cancer remain debated [15].

These studies have suggested that aggressive phenotypes do not always correlate with the clinical outcomes in ER‐positive breast cancer with *BRCA2* PVs. Understanding the link between *BRCA2* deficiency and the ER signaling pathway is essential to clarify the effect of *BRCA2* PVs on the prognosis of ER‐positive breast cancer, which forms the aim of this study. This study examined the expression of ER signaling molecules in breast cancers from *BRCA2* PV carriers and noncarriers. Findings were further validated using ER‐positive breast cancer cell lines with *BRCA2* deficiency.

## 2 Materials and Methods

### 2.1 Molecular Expression Profiling of ESR1 and ERBB2 in BRCA1/2 Mutation‐Associated Breast Cancer

The bc‐GenExMiner database (version 5.1) was used to thoroughly analyze and compare the gene expression levels of *ESR1* and *ERBB2* among patients with *BRCA1/2* mutation‐positive breast cancer and those with sporadic breast cancer. The significance of the observed expression variations was assessed using the Student's *t*‐test.

### 2.2 Patients

A consecutive series of female patients with ER‐positive/HER2‐negative invasive breast carcinomas diagnosed since 2008 were selected. Patients with tumors > 1.0 cm, germline *BRCA2* mutational testing via BRACAnalysis (Myriad Genetics, Salt Lake City, UT, USA) between October 2018 and May 2021, and surgical tumor removal at Osaka University Hospital were included. Conversely, patients who received endocrine therapy before they were diagnosed with breast cancer were excluded.

### 2.3 Immunohistochemistry

Immunohistochemistry (IHC) was conducted as previously described [16]. Formalin‐fixed, paraffin‐embedded tumor samples were obtained from surgical specimens or vacuum‐assisted biopsies (post‐neoadjuvant chemotherapy). Antigen retrieval was performed at pH 6 or 9 and heated to 98°C for 20 or 40 min. Details of the primary antibodies are listed in Table S1. The stained slides were evaluated under a Nikon Eclipse Ci light microscope with a 20 × objective lens (Tokyo, Japan). The image contrast and brightness were adjusted using ImageJ software [17]. Two independent authors (K.K. and I.S.), blinded to the *BRCA2* status, evaluated the IHC images. Each sample was divided as follows; scores 0, 1, 2, and 3 involving 0%, > 0% and ≤ 50%, > 50% and ≤ 90%, and > 90% stained cells, respectively. Scores were evaluated in a hot spot in each sample. When researchers' scores were not consistent, the final scores were determined by discussion.

### 2.4 Statistical Analysis

The baseline characteristics and IHC evaluations were summarized using descriptive statistics. Group differences were analyzed using Fisher's exact test. A two‐sided *p*‐value < 0.05 was considered significant. Analyses were performed using JMP Pro 16 (JMP Statistical Discovery, Cary, NC). Graphs were generated with GraphPad Prism (La Jolla, CA, USA) or Microsoft Excel (Redmond, WA, USA).

### 2.5 Cell Culture

MCF7 cells and their derivatives (American Type Culture Collection, Manassas, VA) were cultured in DMEM/F12 medium (Sigma–Aldrich, St. Louis, MO) supplemented with 10% fetal bovine serum (Sigma–Aldrich). Mycoplasma and other contamination testing were routinely conducted.

### 2.6 Generation of BRCA2‐Deficient Cell Lines

MCF7 cells were transfected with the pCas‐Guide vector containing a *BRCA2*‐targeting guide RNA and linear donor DNA encoding the *puromycin N‐acetyltransferase* gene (Catalog No. KN413464; Origene, Rockville, MD). The guide RNA sequences were 5′‐GGCCTCTCTTTGGATCCAAT‐3′ for vector 1 and 5′‐TAGGACCAATAAGTCTTAAT‐3′ for vector 2. Transfection was performed using TurboFectin 8.0 (Origene) in Opti‐MEM medium (Thermo Fisher Scientific). Two guide RNAs targeting exons 2 and 3 of *BRCA2* were utilized to ensure efficient *BRCA2* disruption. After 48 h of transfection, the cells were cultured in the maintenance medium for 14 days to allow for recovery and expression of the selection marker. Subsequently, the cells were subjected to selection with 1 μg/mL puromycin dihydrochloride (Sigma–Aldrich) for 7–14 days to isolate successfully transfected cells. Individual colonies were manually picked and expanded, and the fastest growing clones were selected for downstream analyses. *BRCA2* deficiency in these clones was confirmed through Western blotting.

### 2.7 Whole‐Exome Sequencing

Genomic DNA was extracted from the parental and *BRCA2*‐deficient cell lines using QIAamp DNA kits (Qiagen, Tokyo, Japan). Libraries were prepared using the Twist Library Preparation Enzymatic Fragmentation Kit + UDIs and the Twist Comprehensive Exome Panel (Twist Bioscience, South San Francisco, CA, USA), following the Twist NGS Workflow protocol. The prepared Illumina libraries were subsequently converted into MGISEQ‐compatible libraries using the MGIEasy Universal Library Conversion Kit (App‐A; MGI, Shenzhen, China). Sequencing was performed on the MGISEQ‐2000RS platform, which generated 100‐bp paired‐end reads. Variants were identified in four steps. First, adapter sequences were trimmed from the raw sequence data using Cutadapt (https://cutadapt.readthedocs.io/en/stable/index.html). Second, the cleaned reads were aligned to the GRCh37/hg19 reference genome using the BWA aligner (https:/bio‐bwa.sourceforge.net/index.shtml). Third, mutations were detected using GATK4's HaplotypeCaller. Fourth, sample‐specific mutations were annotated with ANNOVAR (https://annovar.openbioinfomatics.org/en/latest/). To refine the mutation list, variants were filtered to include only those in coding regions, variants with non‐synonymous mutations, and those with a minor allele frequency < 0.005, excluding common polymorphisms. For mutations specific to *BRCA2*‐deficient cells, additional criteria included a read depth of ≥ 100, a mutational allele frequency between 0.1 and 0.5, and consistent presence in M1‐4 and M2‐6 clones while being absent in the parental MCF7 cells. Whole‐exome sequencing (WES) was performed to characterize genomic alterations in *BRCA2*‐deficient clones as an exploratory analysis.

### 2.8 Whole‐Genome Sequencing

Genomic DNA samples previously used for WES were further analyzed by whole‐genome sequencing (WGS) to achieve comprehensive mutational profiling. Sequencing was conducted on the NovaSeq X Plus platform, generating 151‐bp paired‐end reads. Sequence libraries were prepared using Illumina's PCR‐free library preparation method to minimize amplification bias. Data processing steps, including adapter trimming, sequence alignment, and variant calling, followed the same protocols used in the WES analysis. Within the *BRCA2* genomic region, genomic alterations were descriptively explored in comparison with the parental MCF7 cell line. WGS was conducted to further characterize genomic alterations in *BRCA2*‐deficient clones as an exploratory analysis.

### 2.9 Analysis of AKT and ER Phosphorylation‐Regulated Gene Expression

The expression of eight genes regulated by AKT and ER phosphorylation was analyzed using the cBioPortal platform (https://www.cbioportal.org/). This analysis compared gene expression levels between cases with and without pathogenic *BRCA2* mutations using data derived from the TCGA PanCancer Atlas database. Strict stratification by ER‐positive/HER2‐negative status was not feasible because data on IHC‐based ER and HER2 status were not uniformly available for all cases in the dataset.

### 2.10 Drug Sensitivity Assay

Cells were cultured in 96‐well plates with maintenance medium and treated with either 0.1–5 μM olaparib (Selleck, Yokohama, Japan) or 3–40 μM 4‐hydroxytamoxifen (Abcam, Cambridge, UK) for 7 days. Following treatment, the wells were washed, and the number of adherent cells was quantified using an IN Cell Analyzer 6000 (GE Healthcare, Chicago, IL, USA).

### 2.11 Western Blotting

Protein lysates were prepared, and sodium dodecyl sulfate‐polyacrylamide gel electrophoresis followed by Western blotting was performed as described previously [18]. The primary and secondary antibodies used in these experiments are detailed in Table S1. Protein expression was quantified in ImageJ by measuring the band intensity on the membrane.

## 3 Results

### 3.1 Breast Cancer Subtypes Associated With BRCA1/2 Pathogenic Variants

Using the bc‐GenExMiner database, the expression levels of *ESR1* and *ERBB2* were compared in *BRCA1/2*‐mutated and sporadic breast cancers (Figure S1). *BRCA1*‐mutated cancers exhibited significantly lower expression levels of *ESR1* and *ERBB2* than sporadic breast cancers (*p* < 0.0001). Conversely, *BRCA2*‐mutated cancers showed no significant differences in expression (*p* = 0.6880, *p* = 0.5348). As sporadic breast cancer is characterized by a higher prevalence of ER‐positive, HER2‐negative tumors, these findings confirm that *BRCA2*‐mutated breast cancers predominantly consist of ER‐positive, HER2‐negative subtypes.

### 3.2 Altered ER Signaling Pathway in ER‐Positive/HER2‐Negative Breast Carcinomas With BRCA2 PVs

**FIGURE 1:**
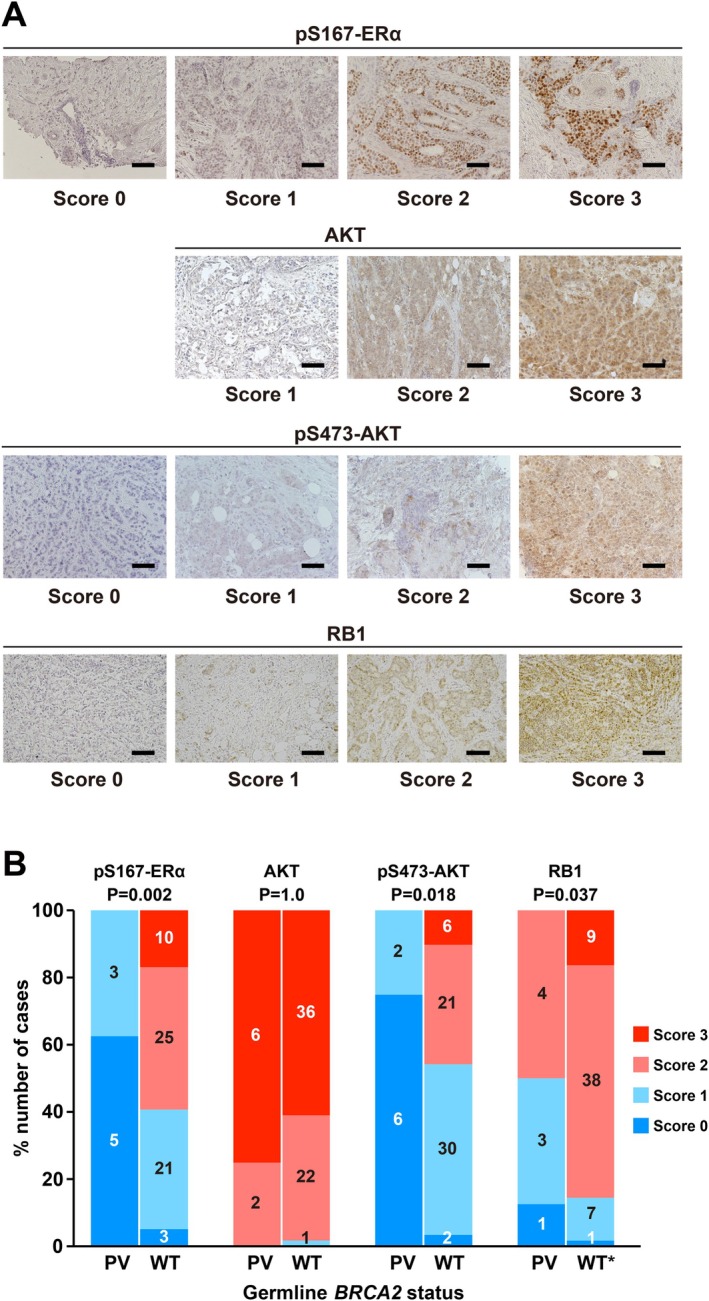
Immunohistochemistry of p‐Ser167 ERα, AKT, p‐Ser473 AKT, and RB1. (A) Representative immunohistochemistry images demonstrating staining intensity scores of 0, 1, 2, and 3 for p‐Ser167 ERα, AKT, p‐Ser473 AKT, and RB1. Scale bar, 100 μm. (B) Statistical summary showing the association between germline *BRCA2* status and immunohistochemical staining intensity. Numbers represent the number of cases within each score category. *p*‐values were calculated using Fisher's exact test by comparing cases with scores of 0–1 versus scores of 2–3. Four cases could not be immunostained due to insufficient material. ER, estrogen receptor; p‐, phosphorylated; PV, pathogenic variant; WT, wild‐type.

The expression levels of proteins involved in the ER signaling pathway in clinical breast cancer samples were examined. A total of 59 patients with *BRCA2‐*wild‐type and 8 with *BRCA2* PVs who underwent BRACAnalysis between October 2018 and May 2021 were identified. The baseline characteristics of the patients were comparable between *BRCA2* PV carriers and noncarriers (Table 1). We focused on key upstream and downstream components of the ER signaling pathway to assess alterations associated with *BRCA2* deficiency. We evaluated AKT and ERα phosphorylation given that AKT is a key upstream kinase that phosphorylates ERα at Ser167, a modification that regulates ER transcriptional activity. We evaluated RB1 as a critical downstream cell cycle regulator and a clinically relevant determinant of response to CDK4/6 inhibitors in ER‐positive breast cancer. IHC staining was performed on ER‐positive/HER2‐negative breast carcinoma samples (Figure 1A). The expression levels of p‐Ser167 ERα, p‐Ser473 AKT, and RB1 were significantly lower in the *BRCA2* PV group than in the *BRCA2*‐wild‐type group (Figure 1B). Immunostaining analysis further indicated the dephosphorylation of AKT and ER in the *BRCA2* PV group. Due to the limited size of the *BRCA2* PV cohort (*n* = 8), the analysis was extended using a comprehensive database. The expression levels of eight genes regulated by AKT and ER phosphorylation were analyzed (Figure S2). The analysis plots depicted the *BRCA2* mutation status on the *x*‐axis and mRNA expression levels on the *y*‐axis. Individual data points are represented as dots, with different colors indicating specific *BRCA2* mutation types. The range is indicated with vertical lines, and the median value is indicated with horizontal lines. The analysis included 994 cases, comprising 966 *BRCA2* wild‐type and 28 *BRCA2‐*mutated cases (including variants of uncertain significance [VUS]). The analysis focused on PV, excluding VUS variants, with truncating mutations (black dots) as the predominant pathogenic variant type, compared with wild‐type cases (light blue dots). No consistent differential expression patterns were observed across the analyzed genes.

**TABLE 1:** Baseline characteristics of patients stratified by germline *BRCA2* status.

Category		*BRCA2* pathogenic variants (*n* = 8)	*BRCA2* wild type (*n* = 59)	*p*
		*n* (%)	*n* (%)	
Menopausal status	Premenopausal	6 (75.0)	39 (66.1)	1.0
	Postmenopausal	2 (25.0)	20 (33.9)	
T status (T1 vs. T2–4)	T1	3 (37.5)	17 (28.8)	0.687
	T2	4 (50.0)	29 (49.2)	
	T3	1 (12.5)	5 (8.5)	
	T4	0 (0)	8 (13.6)	
N status (N0 vs. N1–3)	N0	3 (37.5)	22 (37.3)	1.0
	N1	4 (50.0)	23 (39.0)	
	N2	1 (12.5)	8 (13.6)	
	N3	0 (0)	6 (10.2)	
M status	M0	7 (87.5)	51 (86.4)	1.0
	M1	1 (12.5)	8 (13.6)	
ER	Allred score 7 or 8	7 (87.5)	58 (98.3)	0.226
	Allred score ≤ 6	1 (12.5)	1 (1.7)	
PgR	Negative	1 (12.5)	10 (16.9)	1.0
	Positive	7 (87.5)	49 (83.1)	
Histological grade (Grade 1 and 2 vs. 3)	Grade 1	0 (0)	15 (25.4)	0.669
	Grade 2	7 (87.5)	28 (47.5)	
	Grade 3	1 (12.5)	16 (27.1)	
Pathological type	IDC	7 (87.5)	48 (81.4)	1.0
	Others	1 (12.5)	11 (18.6)	
Ki‐67	≤ 20%	1 (12.5)	23 (39.0)	0.631
	> 20%	3 (37.5)	30 (50.8)	
	Unknown	4 (50.0)	6 (10.2)	

Abbreviations: ER, estrogen receptor; IDC, invasive ductal carcinoma; *p*‐value, Fisher's exact tests; PgR, progesterone receptor.

### 3.3 Generation of BRCA2‐Deficient Cell Lines

**FIGURE 2:**
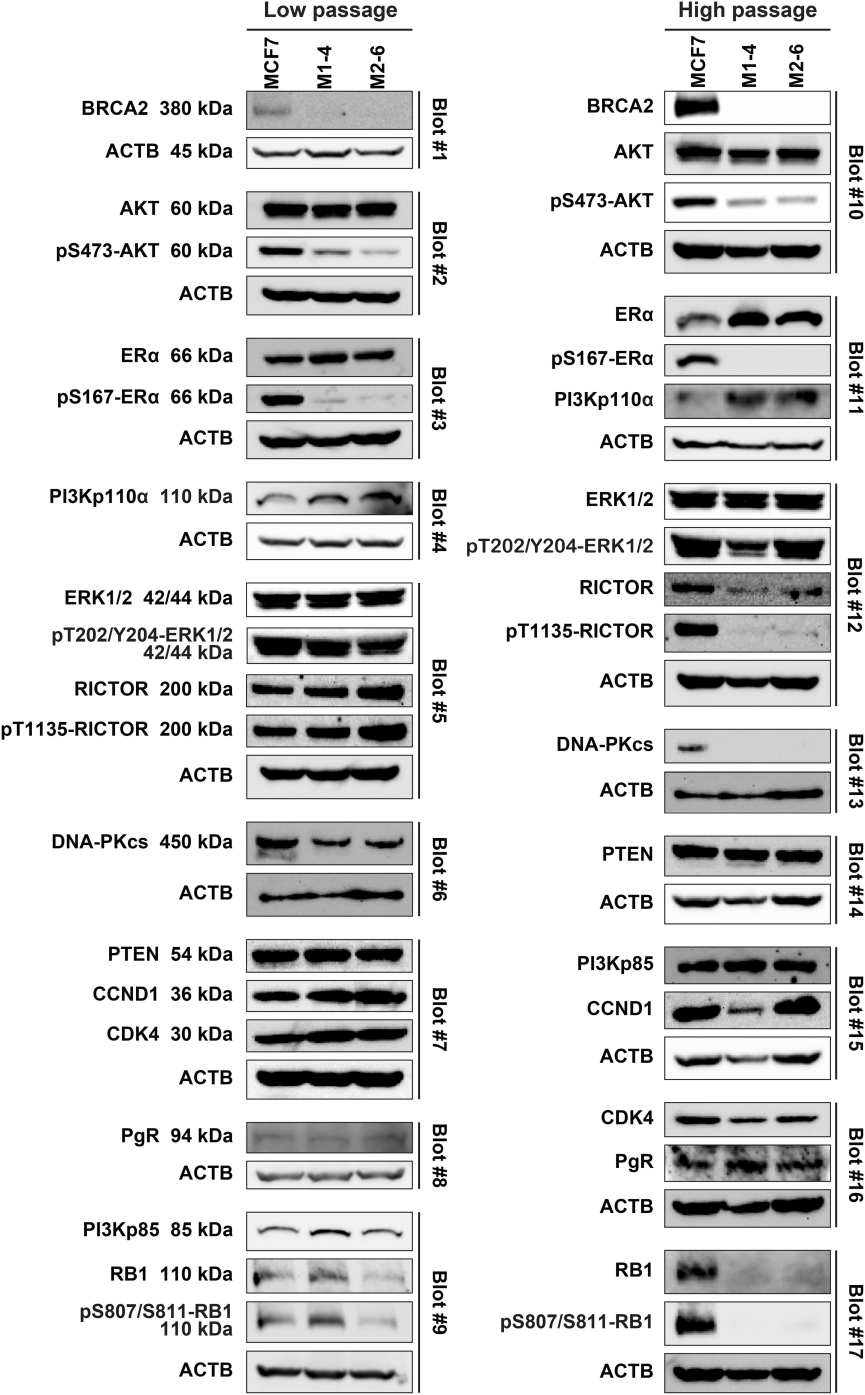
Western blot analysis was performed to evaluate the proteins involved in the estrogen receptor signaling pathway in *BRCA2*‐deficient M1‐4 and M2‐6 cell lines and the parental MCF7 cell line. Protein expression was assessed in two cell batches: Low‐passage (≤ 2 months) and high‐passage (≥ 8 months) for each cell line. Some proteins are not arranged side‐by‐side because the proteins detected from the same membrane were grouped with their respective loading controls.

To validate the clinical findings, ER‐positive/HER2‐negative breast cancer cell lines with *BRCA2* deficiency were generated, and the expression and phosphorylation of proteins involved in the ER signaling pathway were examined. Genome editing was performed on three cell lines (MCF7, T‐47D, and ZR75‐1). Ultimately, two *BRCA2*‐deficient clones (M1‐4 and M2‐6) were successfully generated from MCF7 cells using two distinct guide RNAs. However, *BRCA2‐*deficient cell lines could not be established in T‐47D and ZR75‐1 cells. Western blot analysis confirmed that BRCA2 expression was substantially reduced in M1‐4 and M2‐6 clones (Figure 2, top). For further analysis, two batches of each cell line were prepared: low‐passage cells (≤ 2 months) and high‐passage cells (≥ 8 months). WES was performed on all cell lines, and WGS was conducted on three low‐passage cell lines in exploratory analyses. Although coding‐region variants in *BRCA2* were not identified with WES using the applied filtering criteria (Table S2), intronic variants that were absent in the parental MCF7 cell line were found in both clones with WGS (Table 2).

**TABLE 2:** Mutations identified in the M1‐4 clone or M2‐6 clone that are absent in the parental MCF7 cells.

Clone	Chr	Start	End	Ref	Alt	Func.refGene	AF
M1‐4	13	32 939 247	32 939 247	T	—	Intronic	1
M1‐4	13	32 970 100	32 970 100	—	C	Intronic	1
M2‐6	13	32 939 247	32 939 247	T	—	Intronic	1
M2‐6	13	32 970 085	32 970 085	—	TTTC	Intronic	0.88

Abbreviations: AF, allele frequency; Alt, altered nucleotide(s); Chr, chromosome; Func.refGene, gene function annotation; Ref, reference nucleotide(s); Start/End, nucleotide alteration position.

### 3.4 Altered ER Signaling Pathway in BRCA2‐Deficient MCF7 Cells

The expression levels of various proteins involved in the ER signaling pathway in *BRCA2*‐deficient MCF7 cells were investigated (Figure 2). Comprehensive membrane blots and loading controls from the same membranes are presented in Figures [Link], [Link] because of the large number of Western blot membranes. Although total ERα and AKT protein levels remained consistently high across all cell lines, the phosphorylation of ERα at Ser167 and AKT at Ser473 was remarkably reduced in M1‐4 and M2‐6 clones, regardless of passage (low or high). Protein expression levels were quantified by measuring the band intensity using ImageJ. Protein levels were normalized to their respective loading controls, and relative expression values were calculated using the MCF7 parental cell line as the baseline for *BRCA2*‐deficient cell lines (Table S3). The observed reduction in phosphorylation was not associated with detectable alterations in the corresponding coding regions as determined by WES (Table S2). The ERK1/2 and PI3K‐AKT pathways are crucial for ERα phosphorylation [19]. No consistent changes were observed in the total and the phosphorylated forms of ERK1/2 and the PI3K subunits p85 and p110α in M1‐4 and M2‐6 cells. Furthermore, RICTOR, a core component of mTORC2, showed equivalent expression levels in low‐passage MCF7, M1‐4, and M2‐6 cells. Notably, RICTOR phosphorylation at Thr1135 decreased in high‐passage M1‐4 and M2‐6 cells. Because RICTOR Thr1135 phosphorylation negatively regulates mTORC2 through a feedback mechanism [20], the observed reduction may reflect altered mTORC2 regulation in these *BRCA2*‐deficient cells. Moreover, the DNA‐dependent protein kinase catalytic subunit decreased in high‐passage M1‐4 and M2‐6 clones. In contrast, the expression levels of proteins below the estrogen signaling pathway, including PTEN, PgR, CDK4, and cyclin D1, indicated no consistent differences among MCF7, M1‐4, and M2‐6 cells. However, the tumor‐suppressor RB1 and its phosphorylated form were markedly reduced in high‐passage *BRCA2*‐deficient cells. The relative protein expression values are presented in Table S3.

### 3.5 Effect of BRCA2 Deficiency on the Sensitivity of Olaparib and Tamoxifen

**FIGURE 3:**
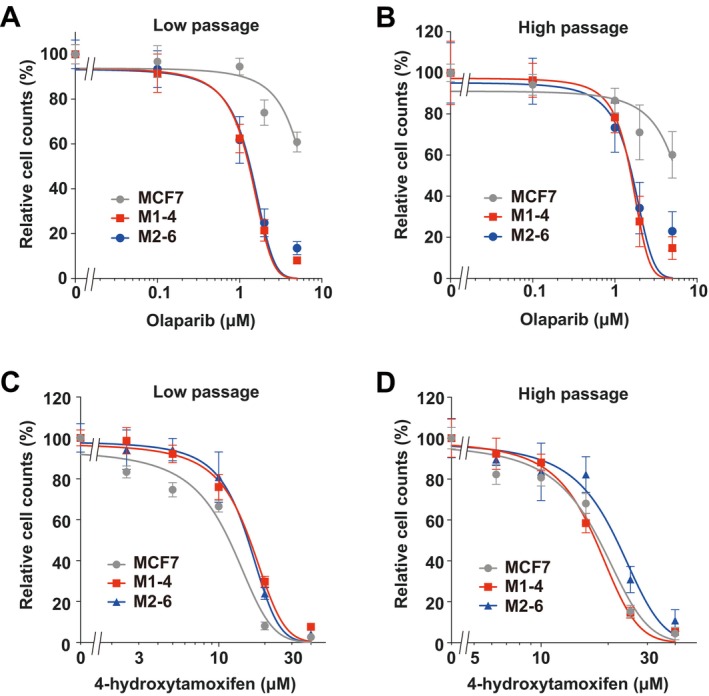
Drug sensitivity of *BRCA2*‐deficient and parental MCF7 cells. Cells were analyzed in two passaged groups: Low‐passage (≤ 2 months) and high‐passage (≥ 8 months). Dose–response curves for olaparib treatment across MCF7, M1‐4, and M2‐6 cells in (A) low‐ and (B) high‐passage groups. Dose–response curves for 4‐hydroxytamoxifen treatment across the same cell lines in (C) low‐ and (D) high‐passage groups.

*BRCA2* deficiency leads to synthetic lethality when the alternative DDR pathway is inhibited [21, 22]. To assess this effect, the sensitivity of MCF7, M1‐4, and M2‐6 cells to the PARP inhibitor olaparib was evaluated (Figure 3A,B). Low‐ and high‐passage batches of the *BRCA2*‐deficient M1‐4 and M2‐6 clones exhibited significantly greater sensitivity to olaparib than the parental MCF7 cells. This increased sensitivity suggests that BRCA2 in M1‐4 and M2‐6 cells was not only quantitatively reduced but also functionally impaired. In addition, the sensitivity of MCF7, M1‐4, and M2‐6 cells to 4‐hydroxytamoxifen was examined to determine whether *BRCA2* deficiency affected the tamoxifen response (Figure 3C,D). Tamoxifen sensitivity was comparable among MCF7, M1‐4, and M2‐6 cells in low‐ and high‐passage batches. These results indicate that *BRCA2* deficiency does not affect tamoxifen sensitivity in MCF7 cells.

## 4 Discussion

This study demonstrated that *BRCA2* deficiency in ER‐positive/HER2‐negative breast cancer impairs the phosphorylation of AKT at Ser473 and ERα at Ser167 in both clinical samples and experimental models. AKT is the primary kinase responsible for phosphorylating ERα at Ser167 [23], and AKT phosphorylation at Ser473 is essential for its full kinase activity [24]. Therefore, the reduced AKT activity in *BRCA2*‐deficient MCF7 cells likely contributed to ERα dephosphorylation at Ser167.

Breast cancer is a heterogeneous disease that includes multiple molecular subtypes with distinct biological characteristics and therapeutic vulnerabilities. In this context, ER‐positive/HER2‐negative tumors are the most prevalent molecular subtype driven primarily by ER signaling. Specifically, ERα phosphorylation at Ser167, which is regulated by growth factor‐associated pathways including the PI3K/AKT axis, can preferentially promote non‐genomic signaling, suggesting that ERα phosphorylation does not always translate into proportional changes in the expression levels of classic estrogen‐responsive genes or proteins [25].

Initial PCR analyses using primers flanking sites targeted by the guide RNAs provided limited information on *BRCA2* gene disruption and were therefore insufficient to provide a definitive genomic‐level assessment. Subsequent real‐time PCR analysis revealed no significant differences in *BRCA2* mRNA levels between the perturbed cells and the parental MCF7 cell line (relative levels: 0.859 and 1.096 in M1‐4 and M2‐6 clones). In contrast, Western blot analysis revealed a consistent decrease in BRCA2 protein expression level, with functional loss supported by the increased sensitivity of *BRCA2*‐deficient clones to the PARP inhibitor olaparib. The discrepancy between the lack of a change in *BRCA2* mRNA levels and the reduced BRCA2 protein expression levels in these clones suggests that BRCA2 deficiency in these cells is mediated at the posttranscriptional or posttranslational level rather than through reduced transcription. Potential mechanisms underlying this finding include altered translation, enhanced protein degradation, and aberrant transcript processing. In this context, we identified intronic variants unique to the *BRCA2*‐deficient clones that were absent in the parental MCF7 cell line with WGS. This finding raised the possibility that abnormal splicing or transcript processing may contribute to reduced BRCA2 protein levels; however, we did not directly examine this hypothesis in the present study. Taken together, these findings support the validity of the *BRCA2*‐deficient cell models used in the present study.

To date, no study has reported a direct association between BRCA2 and AKT, making this the first study to suggest a positive link between BRCA2 and AKT signaling. In contrast, the relationship between BRCA1 and AKT has been previously established. BRCA1 binds to phosphorylated AKT, promoting its degradation through ubiquitination [26], thereby acting as a negative regulator of AKT signaling. Increased AKT phosphorylation in *BRCA1*‐deficient cells leads to chromosomal instability and fosters tumorigenesis in *BRCA1*‐deficient breast epithelial cells [27]. Although BRCA2 mainly functions in DSB repair, unlike the broader functions of BRCA1, it may regulate AKT phosphorylation through a distinct mechanism that warrants further investigation. One hypothesis is that BRCA2, despite lacking direct kinase activity, may be involved in AKT dephosphorylation through major phosphatases such as PP2A and PHLPP. As previously noted, BRCA2 functions mainly as a tumor‐suppressor gene and is involved in DNA repair, particularly in homologous recombination repair. Persistent DDR alterations caused by *BRCA2* deficiency could potentially enhance the activation of PP2A and PHLPP or induce PHLPP modifications as part of a stress response, thereby influencing AKT regulation. Consequently, future studies should investigate the activity of PP2A and PHLPP in *BRCA2*‐deficient cells and evaluate changes in these phosphatases when DDR inhibitors are applied. These studies provide valuable insights into the potential mechanism by which BRCA2 might indirectly regulate AKT signaling via phosphatase modulation.

Although further mechanistic studies, such as those employing pharmacologic AKT inhibition, will be informative in elucidating the direct contribution of AKT activity to ERα Ser167 phosphorylation, detailed mechanistic dissection was considered beyond the scope of the present translational study. Moreover, although further analyses of cellular phenotypes, such as proliferation, apoptosis, and migration, can provide additional insight, the present study focused on therapeutically relevant functional outcomes, including sensitivity to endocrine therapy and PARP inhibition.

The lack of consistent findings in our gene expression analysis using cBioPortal may be attributed to the statistical limitations resulting from the small sample sizes. The TCGA PanCancer Atlas database analysis identified only 12 cases with *BRCA2* mutations, of which just nine carried PV—a cohort size comparable with our study population. With the expected increase in clinical cases in the future, this study aimed to conduct systematic investigations and validate these preliminary observations in subsequent studies.

*BRCA2* deficiency did not significantly affect tamoxifen sensitivity in MCF7 cells. The association between *BRCA2* PVs and endocrine therapy sensitivity remained controversial. *BRCA2*‐deficient ER‐positive breast cancers often exhibit the aggressive luminal B subtype, which could potentially reduce endocrine responsiveness compared to sporadic breast cancers [9]. Some studies have reported poorer breast cancer‐specific outcomes in *BRCA* mutation carriers with ER‐positive breast cancer; however, these studies often analyzed *BRCA1* and *BRCA2* mutations without distinguishing between them [28, 29]. Conversely, several studies focusing specifically on *BRCA2* PV carriers have found no significant association between *BRCA2* PVs and breast cancer prognosis [30, 31, 32, 33]. These findings imply that the effectiveness of endocrine therapy does not differ significantly between *BRCA2* PV carriers and the general population, particularly considering that most *BRCA2* PV‐associated breast cancers are ER‐positive and HER2‐negative. This clinical evidence aligns with our findings, demonstrating similar tamoxifen sensitivity between *BRCA2*‐deficient and *BRCA2*‐wild‐type breast cancer cells.

The influence of AKT phosphorylation at Ser473 and ERα phosphorylation at Ser167 on endocrine therapy sensitivity has been extensively studied. AKT phosphorylation at Ser473 is linked to the activation of the PI3K/AKT/mTOR pathway, which contributes to tamoxifen resistance and poorer overall survival in patients with ER‐positive breast cancer [34, 35, 36]. In contrast, ERα phosphorylation at Ser167 has been suggested to enhance tamoxifen sensitivity, opposing the effect of AKT Ser473 phosphorylation [37, 38]. However, clinically, ERα Ser167 phosphorylation does not correlate with survival outcomes in patients receiving tamoxifen [39]. Supporting this, in a large cohort from the Intergroup Exemestane Study, Szijgyarto et al. did not find a correlation between the phosphorylation status of AKT, MAPK, and ERα and disease‐free survival in patients undergoing endocrine therapy [40]. Collectively, the dephosphorylation of AKT at Ser473 and ERα at Ser167 observed in *BRCA2*‐deficient breast cancer cells likely has minimal impact on tamoxifen sensitivity in clinical settings, consistent with our findings.

Although AKT and ERα dephosphorylation may not directly impact sensitivity to endocrine therapy, our results could be significant when considering molecularly targeted drugs which are used alongside endocrine treatments. Capivasertib, an AKT inhibitor, is effective in treating metastatic and advanced breast cancer cases with *PIK3CA* or *AKT* activation or *PTEN* inactivation [41]. However, its antitumor effect was reduced in breast cancers lacking AKT Ser473 phosphorylation in a mouse patient‐derived xenograft model [42]. Because AKT Ser473 dephosphorylation was observed in *BRCA2*‐deficient ER‐positive breast cancers in this study, *BRCA2* deficiency may influence sensitivity to capivasertib in this context. RB1 expression was lower in *BRCA2* PV‐associated breast tumors than in those with *BRCA2*‐wild‐type tumors. Our cell line experiments confirmed reduced RB1 expression secondary to *BRCA2* deficiency. Co‐deletion of *BRCA2* and *RB1* is common in prostate cancer among *BRCA2* PV carriers owing to the proximity of the *RB1* locus (13q14.2) to *BRCA2* on chromosome 13q13.1 [43]. Although data are limited, concomitant *BRCA2* and *RB1* deletions have also been reported in breast cancer [44]. Notably, *RB1* mutations impair the efficacy of CDK4/6 inhibitors [45], suggesting that PARP inhibitors may be a more suitable adjuvant therapy than CDK4/6 inhibitors for *BRCA2* PV carriers with high‐risk ER‐positive/HER2‐negative breast cancer.

This study has several limitations. First, the biological behavior of established cancer cell lines with CRISPR/Cas9‐mediated *BRCA2* deficiency may differ from that of breast cancer cells in germline *BRCA2* PV carriers. Therefore, these in vitro findings must be carefully extrapolated to clinical settings. Second, the small sample size of patients with *BRCA2* PVs may have introduced bias into our results. Despite these limitations, the consistency between the in vitro and in vivo findings and the marked reduction in ERα Ser167 and AKT Ser473 phosphorylation strengthen the validity of our results. However, validation in larger cohorts is essential.

In summary, this study identified ERα Ser167 and AKT Ser473 dephosphorylation and reduced RB1 expression in ER‐positive/HER2‐negative breast cancer with *BRCA2* PVs. These findings imply the diverse effects of *BRCA2* deficiency on the ER signaling pathway, potentially altering sensitivity to various breast cancer therapies. More studies are necessary to better understand the relationship between *BRCA2* deficiency and the ER signaling pathway.

## 5 Conclusion

This study demonstrates that *BRCA2* deficiency is associated with altered ER signaling in ER‐positive/HER2‐negative breast cancer, providing potential insights for novel therapeutic strategies.

## Author Contributions

**Kaori Kawasaki:** data curation, funding acquisition, formal analysis, project administration, conceptualization, resources, visualization, writing – original draft, writing – review and editing, validation. **Misato Masuyama:** methodology, investigation, data curation, formal analysis, writing – original draft. **Masafumi Shimoda:** data curation, funding acquisition, formal analysis, project administration, conceptualization, resources, visualization, writing – original draft, writing – review and editing, validation. **Ikumi Seto:** investigation, formal analysis. **Chieko Mishima:** formal analysis, resources. **Yoshiaki Sota:** formal analysis, resources. **Kaori Abe:** conceptualization, resources. **Nanae Masunaga:** conceptualization, resources. **Masami Tsukabe:** conceptualization, resources. **Tetsuhiro Yoshinami:** conceptualization, resources. **Tomohiro Miyake:** conceptualization, resources. **Tomonori Tanei:** conceptualization, resources. **Kenzo Shimazu:** conceptualization, resources, supervision.

## Funding

This study was funded in part by the Japan Society for the Promotion of Science (grant no. 20K08980).

## Ethics Statement

Our protocol was approved by the institutional review boards of Osaka University Hospital (Approval no. 14111) and Osaka University (Approval no. G737), and written informed consent was obtained from each patient. The study was conducted in accordance with the Declaration of Helsinki.

## Consent

Consent for the publication of the research data was included in the consent forms and approved by the Institutional Review Board of Osaka University Hospital. Images from tissue specimens of patients are entirely unidentifiable.

## Conflicts of Interest

M.S. received honoraria from Pfizer. N.M. received honoraria from Pfizer and Eli Lilly. T.Y. received honoraria from AstraZeneca, Pfizer, Eli Lilly, and Novartis. T.M. received honoraria from AstraZeneca. K.S. received honoraria from AstraZeneca, Pfizer, Eli Lilly, and Novartis, holds a grant from AstraZeneca for another study, and received support from AstraZeneca for attending meetings. The other authors declare no conflicts of interest.

## Supporting information


**Figure S1:** Comparative analysis of *ESR1* and *ERBB2* expression. The expression values are reported as log_2_‐transformed normalized expression units. Box plots display median values (horizontal line), interquartile ranges (boxes), and ranges (whiskers). ESR1, estrogen receptor 1; ERBB2, Erb‐B2 receptor tyrosine kinase 2.

**Figure S2:** Gene expression analysis in BRCA2 mutation status. The expression levels of eight genes (*BCL2*, *CCND1*, *CSF1*, *CXCL12*, *CTSD*, *MMP9*, *MYC*, and *VEGFA*), regulated by the AKT and ER phosphorylation pathways, were analyzed across different *BRCA2* mutational states. The x‐axis represents the spectrum of *BRCA2* mutations, whereas the y‐axis represents the log‐transformed mRNA expression levels for each gene. In the TCGA PanCancer Atlas dataset, information on immunohistochemistry‐based ER and HER2 status was not available for all cases, precluding strict stratification by ER‐positive/HER2‐negative status. In addition, the number of cases with pathogenic *BRCA2* variants was limited in this dataset, and statistical comparison was not feasible. Therefore, the data are descriptively presented.
BCL2, B‐cell CLL/lymphoma 2; CCND1, cyclin D1; CXCL12, C‐X‐C motif chemokine ligand 12; CSF1, Colony stimulating factor 1; CTSD, cathepsin D; ER, estrogen receptor; MMP9, matrix metallopeptidase 9; MYC, MYC proto‐oncogene, BHLH transcription factor; VEGFA, vascular endothelial growth factor A.

**Figure S3:** Comprehensive Western blot membrane analysis. To detect multiple proteins from a single membrane, the membrane was cut based on molecular weight before incubation with primary antibodies. Complete membrane images are presented alongside loading controls for proteins detected from the same membrane. Phosphorylated proteins were detected first, followed by membrane stripping and the detection of total protein levels. β‐Actin/ACTB was used as the loading control. This figure shows the Western blot results using low‐passage cell lines, demonstrating the expression levels of BRCA2, AKT, pS473‐AKT, ERα, pS167‐ERα, PI3Kp110, RICTOR, pT1135‐RICTOR, ERK1/2, and pT202/Y204‐ERK1/2 along with their corresponding β‐actin controls. Bands detected from the same membrane are presented as a group.

**Figure S4:** This figure shows the Western blot results using low‐passage cell lines, demonstrating the expression of DNA‐PKcs, PTEN, CCND1, CDK4, PgR, RB1, pS807/811‐RB1, and PI3Kp85 along with their corresponding β‐actin controls. Bands detected from the same membrane are presented as a group.

**Figure S5:** This figure shows the Western blot results using high‐passage cell lines, demonstrating the expression of BRCA2, AKT, pS473‐AKT, PI3Kp110, ERα, pS167‐ERα, RICTOR, pT1135‐RICTOR, ERK1/2, pT202/Y204‐ERK1/2, and DNA‐PKcs along with their corresponding β‐actin controls. Bands detected from the same membrane are presented as a group.

**Figure S6:** This figure shows the Western blot results using high‐passage cell lines, demonstrating the expression levels of PTEN, PI3Kp85, CCND1, PgR, CDK4, RB1, and pS807/811‐RB1 along with their corresponding β‐actin controls. Bands detected from the same membrane are presented as a group.

**Table S1:** List of antibodies used for Western blotting and immunohistochemistry.

**Table S2:** List of mutated genes present in the M1‐4 and M2‐6 clones but absent in the parental MCF7 cells.

**Table S3:** Ratios of protein expression levels.

## Data Availability

The data generated in the present study may be requested from the corresponding author. The raw data from WES and WGS generated in the present study may be found in the DDBJ database (https://www.ddbj.nig.ac.jp/index.html) under accession nos. DRA020116.
