# Supplementary figures and images for: Altered Estrogen Receptor Signaling Pathway in BRCA2‐Deficient Estrogen Receptor‐Positive/HER2‐Negative Breast Cancer
Source: Cancer Rep (Hoboken). 2026 Apr 24;9(4):e70558. doi: 10.1002/cnr2.70558 (PMC13109083; doi:10.1002/cnr2.70558)

Low passage

Blot #1

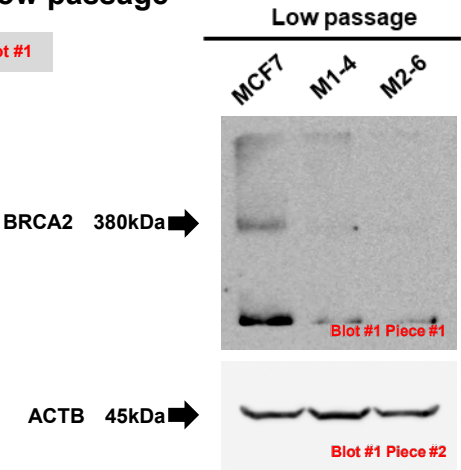

Blot #2

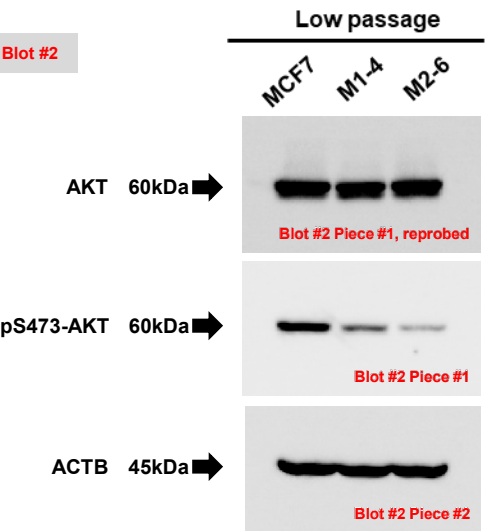

Blot #3

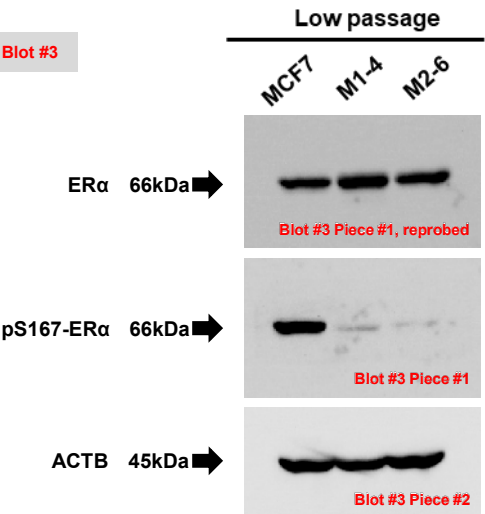

Blot #4

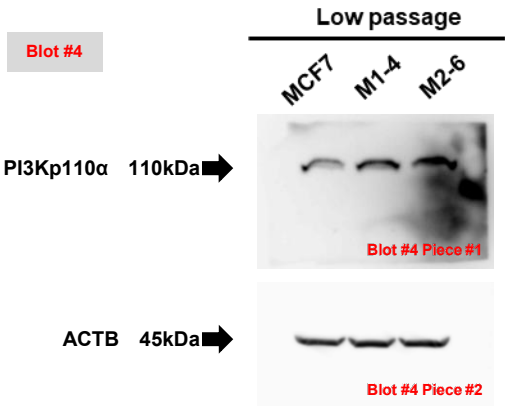

Blot #5

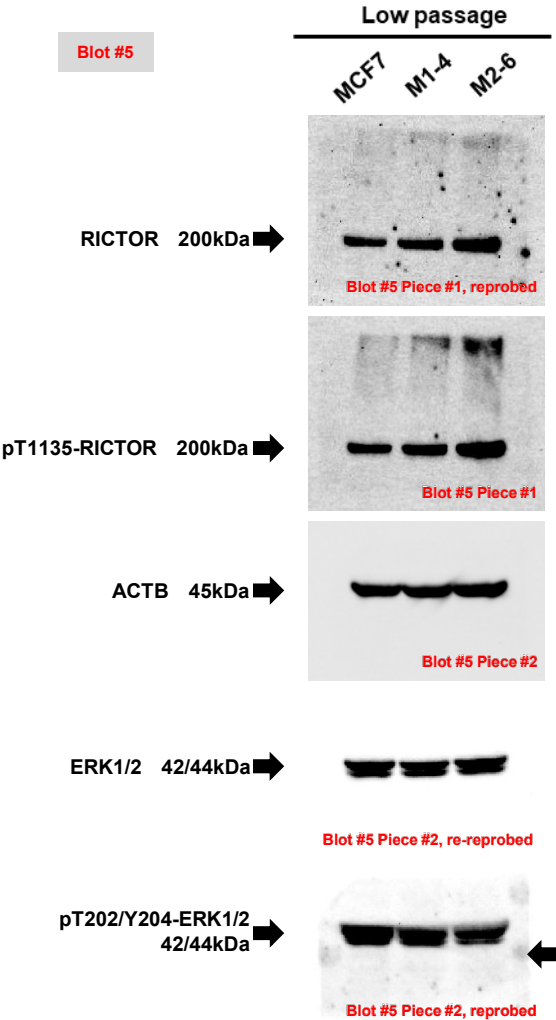

Supplement: Supplementary file 3 — Figure S3: Comprehensive Western blot membrane analysis. To detect multiple proteins from a single membrane, the membrane was cut based on molecular weight before incubation with primary antibodies. Complete membrane images are presented alongside loading controls for proteins detected from the same membrane. Phosphorylated proteins were detected first, followed by membrane stripping and the detection of total protein levels. β‐Actin/ACTB was used as the loading control. This figure shows the Western blot results using low‐passage cell lines, demonstrating the expression levels of BRCA2, AKT, pS473‐AKT, ERα, pS167‐ERα, PI3Kp110, RICTOR, pT1135‐RICTOR, ERK1/2, and pT202/Y204‐ERK1/2 along with their corresponding β‐actin controls. Bands detected from the same membrane are presented as a group. [file CNR2-9-e70558-s005.pdf]
