# Supplementary material for: Altered Estrogen Receptor Signaling Pathway in BRCA2‐Deficient Estrogen Receptor‐Positive/HER2‐Negative Breast Cancer
Source: Cancer Rep (Hoboken). 2026 Apr 24;9(4):e70558. doi: 10.1002/cnr2.70558 (PMC13109083; doi:10.1002/cnr2.70558)
Supplement: Supplementary file 4 — Figure S4: This figure shows the Western blot results using low‐passage cell lines, demonstrating the expression of DNA‐PKcs, PTEN, CCND1, CDK4, PgR, RB1, pS807/811‐RB1, and PI3Kp85 along with their corresponding β‐actin controls. Bands detected from the same membrane are presented as a group. [file CNR2-9-e70558-s007.pdf]

Low passage

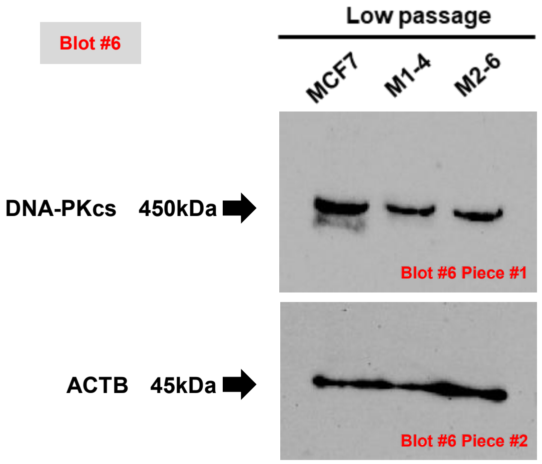

Blot #6
Low passage
MCF7
M1-4
M2-6
DNA-PKcs 450kDa
Blot #6 Piece #1
ACTB 45kDa
Blot #6 Piece #2

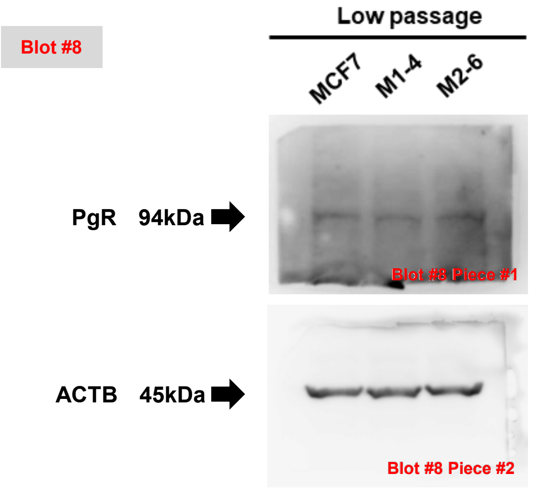

Blot #8
Low passage
MCF7
M1-4
M2-6
PgR 94kDa
Blot #8 Piece #1
ACTB 45kDa
Blot #8 Piece #2

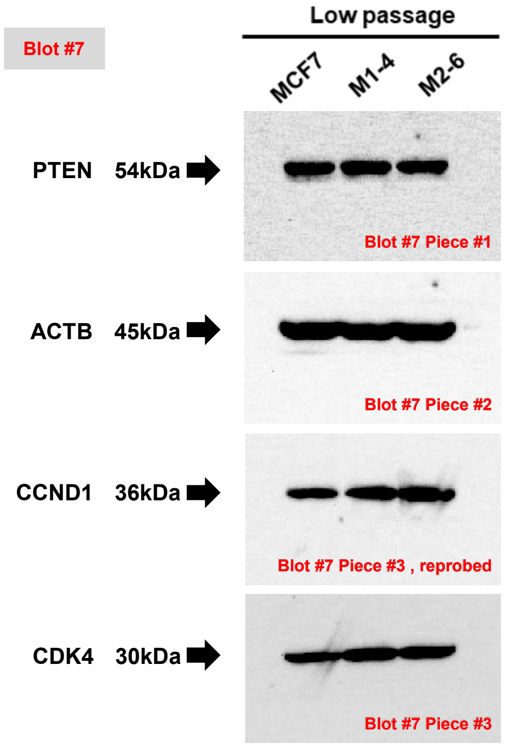

Blot #7
Low passage
MCF7
M1-4
M2-6
PTEN 54kDa
Blot #7 Piece #1
ACTB 45kDa
Blot #7 Piece #2
CCND1 36kDa
Blot #7 Piece #3 , reprobed
CDK4 30kDa
Blot #7 Piece #3

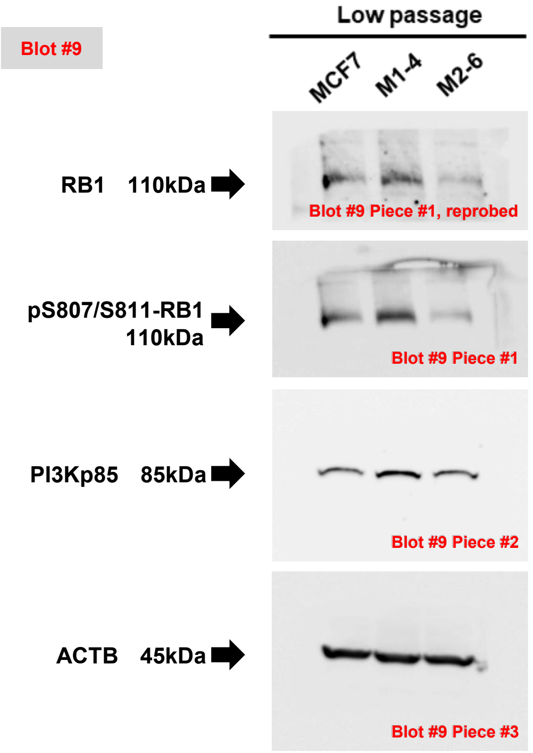

Blot #9
Low passage
MCF7
M1-4
M2-6
RB1 110kDa
Blot #9 Piece #1, reprobed
pS807/S811-RB1
110kDa
Blot #9 Piece #1
PI3Kp85 85kDa
Blot #9 Piece #2
ACTB 45kDa
Blot #9 Piece #3
