# Supplementary material for: Altered Estrogen Receptor Signaling Pathway in BRCA2‐Deficient Estrogen Receptor‐Positive/HER2‐Negative Breast Cancer
Source: Cancer Rep (Hoboken). 2026 Apr 24;9(4):e70558. doi: 10.1002/cnr2.70558 (PMC13109083; doi:10.1002/cnr2.70558)

High passage

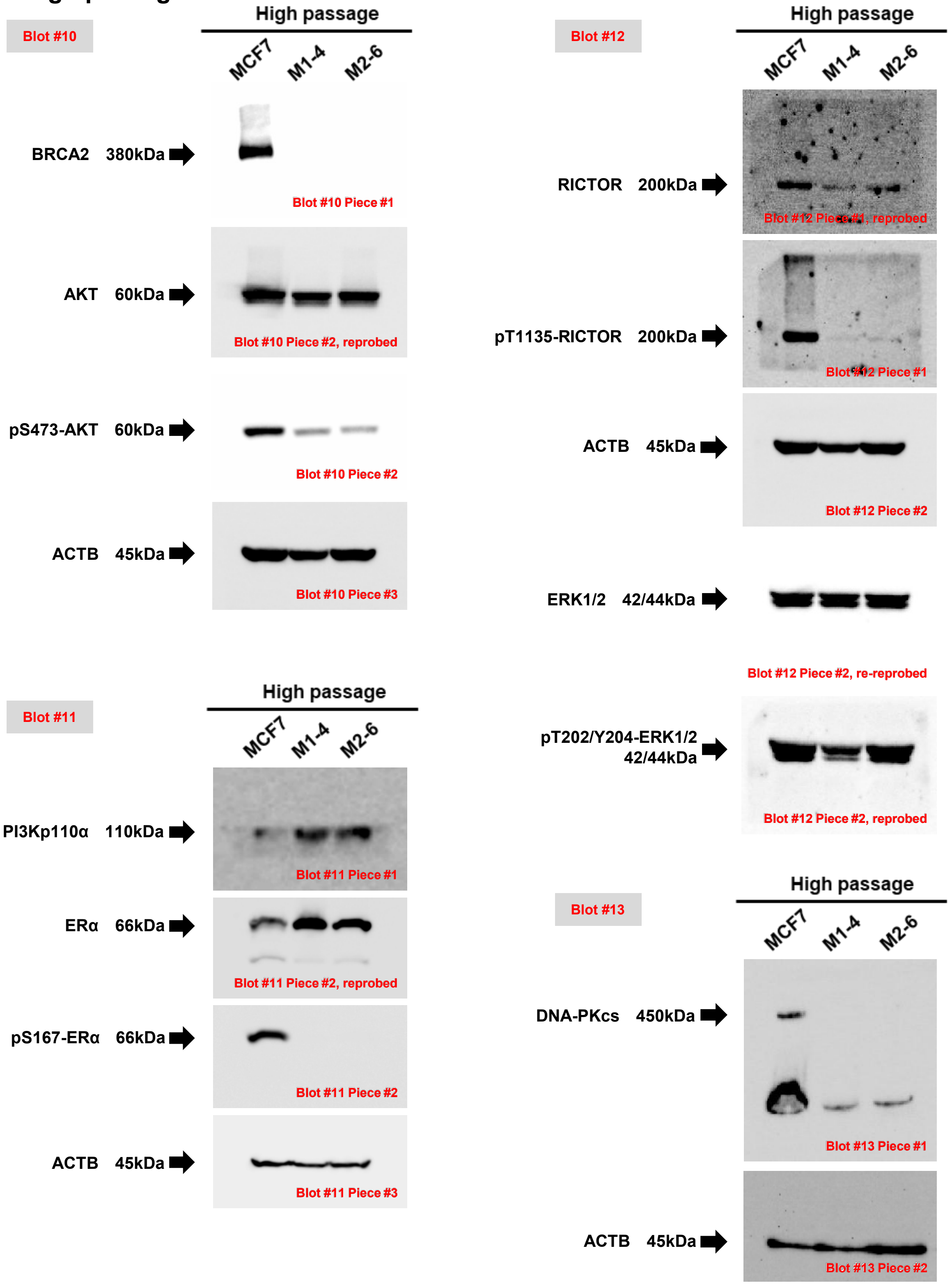

Supplement: Supplementary file 5 — Figure S5: This figure shows the Western blot results using high‐passage cell lines, demonstrating the expression of BRCA2, AKT, pS473‐AKT, PI3Kp110, ERα, pS167‐ERα, RICTOR, pT1135‐RICTOR, ERK1/2, pT202/Y204‐ERK1/2, and DNA‐PKcs along with their corresponding β‐actin controls. Bands detected from the same membrane are presented as a group. [file CNR2-9-e70558-s003.pdf]
