# Supplementary material for: Altered Estrogen Receptor Signaling Pathway in BRCA2‐Deficient Estrogen Receptor‐Positive/HER2‐Negative Breast Cancer
Source: Cancer Rep (Hoboken). 2026 Apr 24;9(4):e70558. doi: 10.1002/cnr2.70558 (PMC13109083; doi:10.1002/cnr2.70558)

High passage

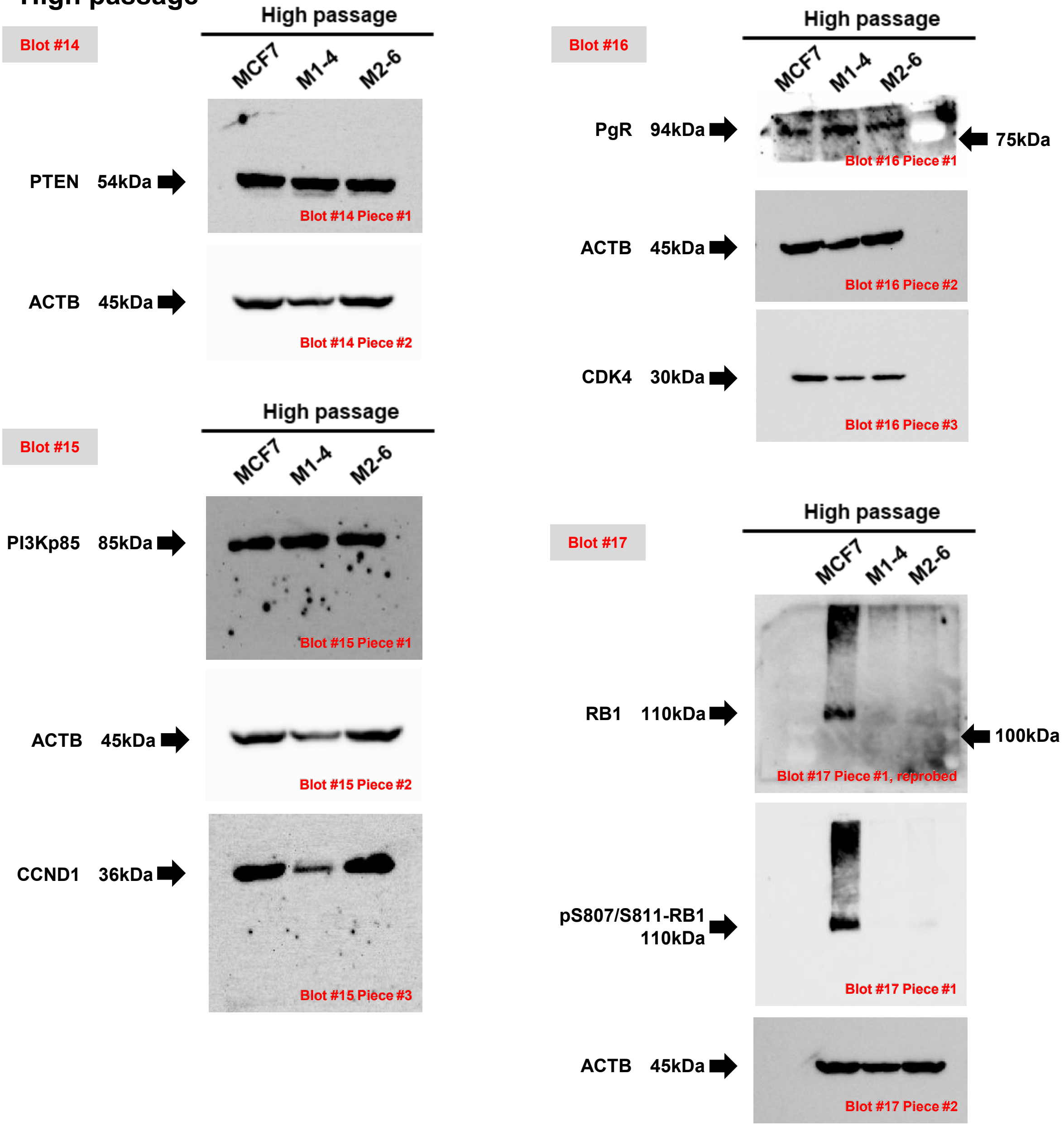

Supplement: Supplementary file 6 — Figure S6: This figure shows the Western blot results using high‐passage cell lines, demonstrating the expression levels of PTEN, PI3Kp85, CCND1, PgR, CDK4, RB1, and pS807/811‐RB1 along with their corresponding β‐actin controls. Bands detected from the same membrane are presented as a group. [file CNR2-9-e70558-s001.pdf]
