# Supplementary material for: Altered Estrogen Receptor Signaling Pathway in BRCA2‐Deficient Estrogen Receptor‐Positive/HER2‐Negative Breast Cancer
Source: Cancer Rep (Hoboken). 2026 Apr 24;9(4):e70558. doi: 10.1002/cnr2.70558 (PMC13109083; doi:10.1002/cnr2.70558)
Supplement: Supplementary file 7 — Table S1: List of antibodies used for Western blotting and immunohistochemistry. [file CNR2-9-e70558-s006.docx]

Table SI. List of antibodies used for Western blotting and immunohistochemistry.

Primary antibodies for Western blotting

| Antigen | Company | Clone | Catalog no. |
| --- | --- | --- | --- |
| BRCA2 | R&D Systems | 234403 | MAB2476 |
| ERα | Abcam | EPR4097 | Ab108398 |
| phosho-Ser167 ERα | Cell Signaling Technology | D5W3Z | 64508 |
| ERK1/2 | Cell Signaling Technology | 137F5 | 4695 |
| phosho-Thr202/Tyr204 ERK1/2 | Cell Signaling Technology | D13.14.4E | 4370 |
| PI3Kp85 | Cell Signaling Technology | 19H8 | 4257 |
| PI3Kp110α | Cell Signaling Technology | C73F8 | 4249 |
| AKT | Cell Signaling Technology | C67E7 | 4691 |
| phosho-Ser473 AKT | Cell Signaling Technology | D9E | 4060 |
| PTEN | Cell Signaling Technology | D4.3 | 9188 |
| PgR | Abcam | PR-AT 4.14 | Ab2764 |
| CDK4 | Cell Signaling Technology | D9G3E | 12790 |
| CCND1 | Cell Signaling Technology | 92G2 | 2978 |
| RB1 | Cell Signaling Technology | D20 | 9313 |
| phosho-Ser807/Ser811 RB1 | Cell Signaling Technology | D20B12 | 8516 |
| RICTOR | Cell Signaling Technology | 53A2 | 2114 |
| phosho-Thr1135 RICTOR | Cell Signaling Technology | D30A3 | 3806 |
| DNA-PKcs | Cell Signaling Technology | E6U3A | 38168 |
| ACTB | Cell Signaling Technology | D6A8 | 8457 |

Secondary antibodies for Western blotting

| Antigen | Company | Catalog no. |
| --- | --- | --- |
| Mouse IgG | Jackson ImmunoResearch | 715-036-151 |
| Rabbit IgG | Cell Signaling Technology | 7074 |

Primary antibodies for immunohistochemistry

| Antigen | Company | Clone | Catalog no. |
| --- | --- | --- | --- |
| phosho-Ser167 ERα | Cell Signaling Technology | D5W3Z | 64508 |
| AKT | Cell Signaling Technology | C67E7 | 4691 |
| phosho-Ser473 AKT | Cell Signaling Technology | D9E | 4060 |
| RB1 | Cell Signaling Technology | 4H1 | 9309 |
