# Supplementary material for: Altered Estrogen Receptor Signaling Pathway in BRCA2‐Deficient Estrogen Receptor‐Positive/HER2‐Negative Breast Cancer
Source: Cancer Rep (Hoboken). 2026 Apr 24;9(4):e70558. doi: 10.1002/cnr2.70558 (PMC13109083; doi:10.1002/cnr2.70558)
Supplement: Supplementary file 8 — Table S2: List of mutated genes present in the M1‐4 and M2‐6 clones but absent in the parental MCF7 cells. [file CNR2-9-e70558-s008.docx]

Table SII. List of mutated genes present in the M1-4 and M2-6 clones but absent in the parental MCF7 cells.

| Chr | Start | End | Ref | Alt | Gene.refGene | ExonicFunc.refGene | DP | Ref | MAF = (DP-Ref)/DP | Description |
| --- | --- | --- | --- | --- | --- | --- | --- | --- | --- | --- |
| 1 | 34286119 | 34286119 | G | T | CSMD2 | nonsynonymous SNV | 117 | 73 | 0.376068376 | CUB and Sushi multiple domains 2 |
| 3 | 27333034 | 27333034 | - | C | NEK10 | frameshift insertion | 267 | 215 | 0.194756554 | NIMA related kinase 10 |
| 3 | 36527661 | 36527661 | C | G | STAC | nonsynonymous SNV | 245 | 202 | 0.175510204 | SH3 and cysteine rich domain |
| 3 | 43122215 | 43122215 | A | T | POMGNT2 | nonsynonymous SNV | 264 | 193 | 0.268939394 | protein O-linked mannose N-acetylglucosaminyltransferase 2 |
| 3 | 140998258 | 140998258 | G | A | PXYLP1 | nonsynonymous SNV | 349 | 280 | 0.197707736 | 2-phosphoxylose phosphatase 1 |
| 3 | 153994688 | 153994688 | T | A | DHX36 | nonsynonymous SNV | 245 | 199 | 0.187755102 | DEAH-box helicase 36 |
| 3 | 183695342 | 183695342 | G | A | ABCC5 | nonsynonymous SNV | 181 | 134 | 0.259668508 | ATP binding cassette subfamily C member 5 |
| 4 | 119952646 | 119952646 | T | C | SYNPO2 | nonsynonymous SNV | 240 | 154 | 0.358333333 | synaptopodin 2 |
| 4 | 128938582 | 128938582 | G | T | ABHD18 | nonsynonymous SNV | 202 | 147 | 0.272277228 | abhydrolase domain containing 18 |
| 5 | 79747475 | 79747475 | G | C | ZFYVE16 | nonsynonymous SNV | 274 | 203 | 0.259124088 | zinc finger FYVE-type containing 16 |
| 6 | 27925728 | 27925728 | C | T | OR2B6 | nonsynonymous SNV | 265 | 218 | 0.177358491 | olfactory receptor family 2 subfamily B member 6 |
| 6 | 51917970 | 51917970 | G | T | PKHD1 | nonsynonymous SNV | 274 | 212 | 0.226277372 | ciliary IPT domain containing fibrocystin/polyductin |
| 6 | 131900380 | 131900380 | T | A | ARG1 | nonsynonymous SNV | 196 | 128 | 0.346938776 | arginase 1 |
| 7 | 105254802 | 105254804 | GAG | - | ATXN7L1 | nonframeshift deletion | 467 | 410 | 0.122055675 | ataxin 7 like 1 |
| 9 | 17466757 | 17466757 | G | T | CNTLN | nonsynonymous SNV | 179 | 120 | 0.329608939 | centlein |
| 9 | 96097867 | 96097871 | GAGGT | - | C9orf129 | frameshift deletion | 230 | 170 | 0.260869565 | pseudogene |
| 9 | 131857711 | 131857711 | C | A | CRAT | nonsynonymous SNV | 275 | 195 | 0.290909091 | carnitine O-acetyltransferase |
| 11 | 77594917 | 77594917 | G | T | INTS4 | nonsynonymous SNV | 281 | 224 | 0.202846975 | integrator complex subunit 4 |
| 12 | 53553905 | 53553905 | G | A | CSAD | nonsynonymous SNV | 293 | 223 | 0.23890785 | cysteine sulfinic acid decarboxylase |
| 12 | 130185085 | 130185085 | C | T | TMEM132D | nonsynonymous SNV | 262 | 179 | 0.316793893 | transmembrane protein 132D |
| 16 | 89613073 | 89613073 | G | C | SPG7 | nonsynonymous SNV | 265 | 223 | 0.158490566 | paraplegin, mitochondrial metlloprotease |
| 17 | 72999640 | 72999640 | C | T | CDR2L | nonsynonymous SNV | 323 | 257 | 0.204334365 | cerebellar degeneration related protein 2 like |
| 19 | 56029228 | 56029228 | G | C | SSC5D | nonsynonymous SNV | 220 | 151 | 0.313636364 | scavenger receptor cysteine rich family member with 5 domains |
| 19 | 57335913 | 57335913 | T | A | PEG3 | nonsynonymous SNV | 306 | 237 | 0.225490196 | paternally expressed 3 |
| 20 | 20616135 | 20616137 | AAG | - | RALGAPA2 | nonframeshift deletion | 277 | 189 | 0.317689531 | Ral GTPase activating protein catalytic subunit alpha 2 |
| 20 | 25258969 | 25258969 | G | T | PYGB | nonsynonymous SNV | 211 | 170 | 0.194312796 | glycogen phosphorylase b |
| 22 | 46627938 | 46627938 | C | G | PPARA | nonsynonymous SNV | 175 | 90 | 0.485714286 | ppar-alpha |
| X | 57935982 | 57935982 | G | T | ZXDA | nonsynonymous SNV | 349 | 225 | 0.35530086 | zinc finger X-linked duplicated A |
| X | 108684674 | 108684674 | G | T | GUCY2F | stopgain | 311 | 237 | 0.237942122 | guanylate cyclase 2F, retinal |
| X | 142717244 | 142717244 | C | T | SLITRK4 | nonsynonymous SNV | 506 | 383 | 0.243083004 | SLIT and NTRK like family member 4 |
| X | 147744036 | 147744036 | G | T | AFF2 | nonsynonymous SNV | 457 | 331 | 0.27571116 | ALF transcription elongation factor 2 |

Alt, altered nucleotide(s); Chr, chromosome; DP, read depth at this position; ExonicFunc.refGene, type of alteration in RefSeq; Gene.refGene, RefSeq gene nomenclature; MAF, mutational allele frequency Ref, reference base(s); Ref, reference sequence nucleotide(s); Start/End, location of nucleotide alteration. Alt, altered nucleotide(s)
