# Supplementary material for: Altered Estrogen Receptor Signaling Pathway in BRCA2‐Deficient Estrogen Receptor‐Positive/HER2‐Negative Breast Cancer
Source: Cancer Rep (Hoboken). 2026 Apr 24;9(4):e70558. doi: 10.1002/cnr2.70558 (PMC13109083; doi:10.1002/cnr2.70558)
Supplement: Supplementary file 9 — Table S3: Ratios of protein expression levels. [file CNR2-9-e70558-s004.docx]

Table SIII. Ratios of protein expression levels.

| Protein | Low passage | | | High passage | | | |
| --- | --- | --- | --- | --- | --- | --- | --- |
|  | MCF7 | M1-4 | M2-6 | MCF7 | M1-4 | M2-6 |  |
| ERα | 1 | 1.5 | 1.1 | 1 | 2.2 | 1.7 |  |
| pS167-ERα | 1 | 0.21 | 0.068 | 1 | 0.018 | 0.013 |  |
| pS167-ERα/ERα | 1 | 0.14 | 0.062 | 1 | 0.0082 | 0.0076 |  |
| AKT | 1 | 0.93 | 1.0 | 1 | 0.97 | 0.95 |  |
| pS473-AKT | 1 | 0.41 | 0.18 | 1 | 0.39 | 0.25 |  |
| pS473-AKT/AKT | 1 | 0.44 | 0.18 | 1 | 0.40 | 0.26 |  |
| RB1 | 1 | 1.0 | 0.26 | 1 | 0.27 | 0.26 |  |
| pS807 + S811-RB1 | 1 | 1.3 | 0.35 | 1 | 0.030 | 0.017 |  |

Protein expression levels were normalized to ACTB expression and relative values calculated by setting the MCF7 cell line expression as 1.
